# Voluntary exercise normalizes the proteomic landscape in muscle and brain and improves the phenotype of progeroid mice

**DOI:** 10.1111/acel.13029

**Published:** 2019-09-06

**Authors:** Jaime M. Ross, Giuseppe Coppotelli, Rui M. Branca, Kyung M. Kim, Janne Lehtiö, David A. Sinclair, Lars Olson

**Affiliations:** ^1^ Department of Neuroscience, Biomedicum Karolinska Institutet Stockholm Sweden; ^2^ Department of Genetics Blavatnik Institute, Paul F. Glenn Center for Biology of Aging Research at Harvard Medical School Boston MA USA; ^3^ Department of Oncology‐Pathology, Science for Life Laboratory Karolinska Institutet Stockholm Sweden

**Keywords:** aging, exercise, mitochondria, mtDNA, PolG, proteomics

## Abstract

The accumulation of mitochondrial DNA (mtDNA) mutations is a suspected driver of aging and age‐related diseases, but forestalling these changes has been a major challenge. One of the best‐studied models is the prematurely aging mtDNA mutator mouse, which carries a homozygous knock‐in of a proofreading deficient version of the catalytic subunit of mtDNA polymerase‐γ (*PolgA*). We investigated how voluntary exercise affects the progression of aging phenotypes in this mouse, focusing on mitochondrial and protein homeostasis in both brain and peripheral tissues. Voluntary exercise significantly ameliorated several aspects of the premature aging phenotype, including decreased locomotor activity, alopecia, and kyphosis, but did not have major effects on the decreased lifespan of mtDNA mutator mice. Exercise also decreased the mtDNA mutation load. In‐depth tissue proteomics revealed that exercise normalized the levels of about half the proteins, with the majority involved in mitochondrial function and nuclear–mitochondrial crosstalk. There was also a specific increase in the nuclear‐encoded proteins needed for the tricarboxylic acid cycle and complex II, but not in mitochondrial‐encoded oxidative phosphorylation proteins, as well as normalization of enzymes involved in coenzyme Q biosynthesis. Furthermore, we found tissue‐specific alterations, with brain coping better as compared to muscle and with motor cortex being better protected than striatum, in response to mitochondrial dysfunction. We conclude that voluntary exercise counteracts aging in mtDNA mutator mice by counteracting protein dysregulation in muscle and brain, decreasing the mtDNA mutation burden in muscle, and delaying overt aging phenotypes.

## 1 INTRODUCTION

A large proportion of humans, as well as laboratory mice kept in standard size cages, lack sufficient daily physical activity and benefit from planned exercise. While effects are noted at different ages, one particularly interesting aspect is the effect of exercise during aging. The hypothesis that an increasing burden of mitochondrial DNA (mtDNA) mutations contributes to aging is supported by the premature, but otherwise strikingly typical, aging phenotype and premature death of “mtDNA mutator mice.” These mice carry a mutation in the mtDNA polymerase PolgA, causing impaired proofreading of mtDNA replication, which results in a markedly increasing mtDNA mutation load throughout life (Kauppila, Kauppila, & Larsson, 2017; Trifunovic et al., 2004). Genetically, very similar “mutator mice” have a very similar phenotype (Kujoth et al., 2005). In the human PolG gene, close to 200 specific mutations have been identified. They are coupled to a large number of different rare diseases, affecting eyes, liver, brain, muscle, fertility, and more (Mitochondrial DNA Replication Group at NIEHS: http://tools.niehs.nih.gov/polg/) showing the crucial role of PolG. To generate the mtDNA mutator mouse, a replacement of aspartate by alanine (D257A) was made in the proofreading exonuclease domain of mouse PolG, severely impairing polymerase proofreading of mtDNA, leading to increased mtDNA mutations in all cells and a general aging‐like premature aging phenotype (Trifunovic et al., 2004). The mice have been used to model a worst‐case scenario of mtDNA mutation load and to study possible effects of treatments, including forced treadmill exercise (Clark‐Matott et al., 2015; Safdar et al., 2011, 2016), high‐fat diet (Wall et al., 2015), and caloric restriction (Someya et al., 2017), as well as overexpression of proteins involved in mitochondrial biogenesis, such as the peroxisome proliferator‐activated receptor γ coactivator‐1α (PGC‐1α) (Dillon et al., 2012) and drug treatments (Shabalina et al., 2017). Overall, exercise has been shown to have the most beneficial effects with respect to behavior and other observable phenotypes, as well as regarding metabolic and biochemical parameters. Remarkably, forced exercise has also been reported to decrease the mtDNA mutation load of mutator mice in skeletal muscle (Safdar et al., 2011, 2016).

To address factors underlying aging due to a decline in mitochondrial function, and the effects of exercise during aging, we have profiled proteins in brain and muscle of sedentary and exercised mtDNA mutator mice. To minimize possible interfering effects of stress associated with forced treadmill running maintained by electric stimuli (Safdar et al., 2011), we selected to improve social contacts and to use voluntary exercise. This was accomplished by providing pairs of mice with running wheels in their home cages. We found that voluntary exercise improves spontaneous activity and counteracts visible aging phenotypes without any major alterations in lifespan, as well as normalizes many, but not all protein alterations found in skeletal muscle and brain tissue from mtDNA mutator mice. Voluntary exercise also decreases the mtDNA mutation load and may have facilitated some of the observed improvements in protein levels, phenotypes, and behavior. Interestingly, we found cerebral cortex is better protected from mitochondrial dysfunction compared to striatum, and the striking improvements observed with exercise in mtDNA mutator mice to be correlated with normalized levels of the nuclear‐encoded proteins involved in mitochondrial quality control, complex II, the tricarboxylic acid (TCA) cycle, and coenzyme Q biosynthesis.

## 2 RESULTS

**Figure 1:**
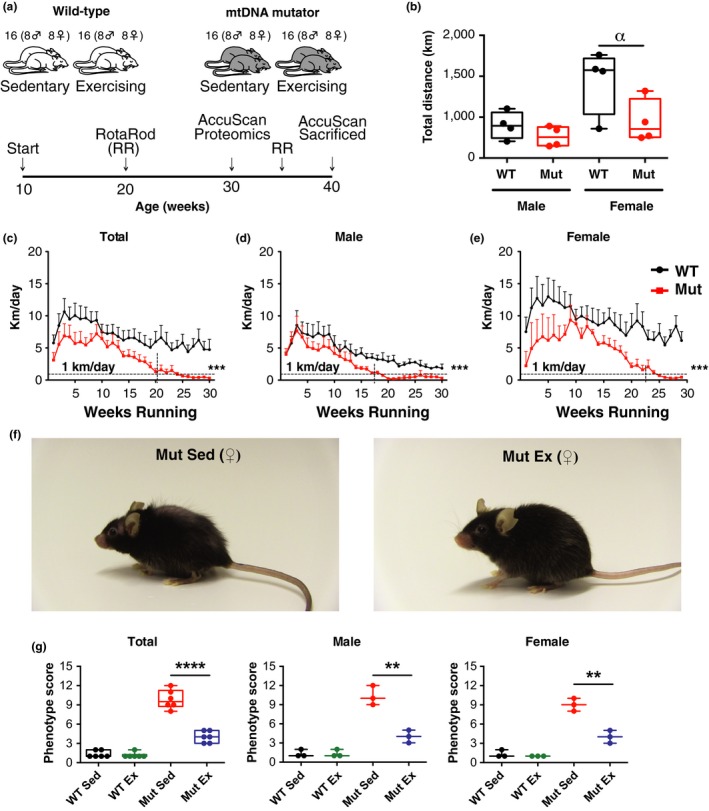
Experimental setup, running behavior, and effects of exercise on mtDNA mutator mouse phenotype. (a) Experimental setup with mtDNA mutator (MUT) and wild‐type (WT) littermates. Not shown: Seven females from the sedentary and exercising mtDNA mutator group were used in the longevity study. (b) Total distance run by male and female WT (black, *N* = 16) and Mut (red, *N* = 16) mice housed in pairs. (c–e) Running behavior of WT (black, *N* = 16) and Mut (red, *N* = 16) mice using the home cage running wheels, shown as total, and separately for males and females. (b–e) Note: This is the combined running of two individuals/cage. (f) Representative picture of thirty‐five‐week‐old sedentary (Mut Sed) and exercising (Mut Ex) mtDNA mutator mice (females are shown). Mut Ex mice present with fewer signs of premature aging phenotypes, such as alopecia, kyphosis, and reduction in body size, as compared to Mut Sed mice. (g) Twelve‐point phenotypic aging score in 35‐week‐old sedentary and exercising WT and mtDNA mutator mice shown as total (*N* = 6 for each group), and separately for males (*N* = 3 per group) and females (*N* = 3 per group). Significances were determined by unpaired *t* test or two‐way ANOVA with post hoc analysis with α *p* < .10, *p < .05, **p < .01, ***p < .001, ****p < .0001

In order to determine how voluntary exercise affects different tissues of the mtDNA mutator mouse at a proteomic level, equal numbers of male and female WT and mtDNA mutator mice (*n* = 16 per condition) were caged by two and provided with one running wheel at the age of 10 weeks. Motor coordination and locomotor activity were tested and compared to equal numbers of sedentary mutator and WT mice at different ages using rotarod and accuscan box tests, respectively (Figure 1a).

### 2.1 PolG genotype and sex differences of voluntary exercise performance

Wild‐type mice ran significantly more than mtDNA mutator mice during the 30 weeks of training (1,302 ± 271.2 km vs. 570.9 ± 83.6 km) (Figure 1b and c). Running activity in both groups peaked between the 3rd and 9th week and then declined, as also seen in other studies (McMullan et al., 2016; Suwa et al., 2016; Figure 1c). Combining sexes, the WT mice ran more than mtDNA mutator mice during all 30 weeks. Dividing by sex, we found that both WT and Mut females ran more than males and that the overall fitness of mtDNA mutator females declined slower than that of mtDNA mutator males. Thus, females begin to run <1 km/day around 34 weeks of age, while males fall below the 1 km/day level already at 27 weeks of age (Figure 1d and e). The circadian rhythm was maintained in running mtDNA mutator mice as demonstrated by the fact that both WT and mtDNA mutator mice adhered to the expected diurnal activity pattern, using the running wheels at night. However, we observed that mtDNA mutator mice ran for a shorter period of the night, suggesting less endurance as compared to WT mice (Figure S1).

### 2.2 Voluntary exercise improves the phenotype of mtDNA mutator mice

Using our phenotype score to assess the signs of premature aging (Ross et al., 2013), we found male and female WT sedentary and WT exercised mice to have a normal adult appearance (score < 2), while sedentary male and female mtDNA mutator mice at 35 weeks had marked signs of aging (score ≈9–10 on the 0–12 scale). Exercise significantly reduced the aging scores of mtDNA mutator males and females (score ≈4, *p* < .001). Hence, the visible aging phenotypes were markedly counteracted by exercise in both sexes, but not fully normalized (Figure 1f and g).

### 2.3 Voluntary exercise improves locomotor behaviors of WT and mtDNA mutator mice

**Figure 2:**
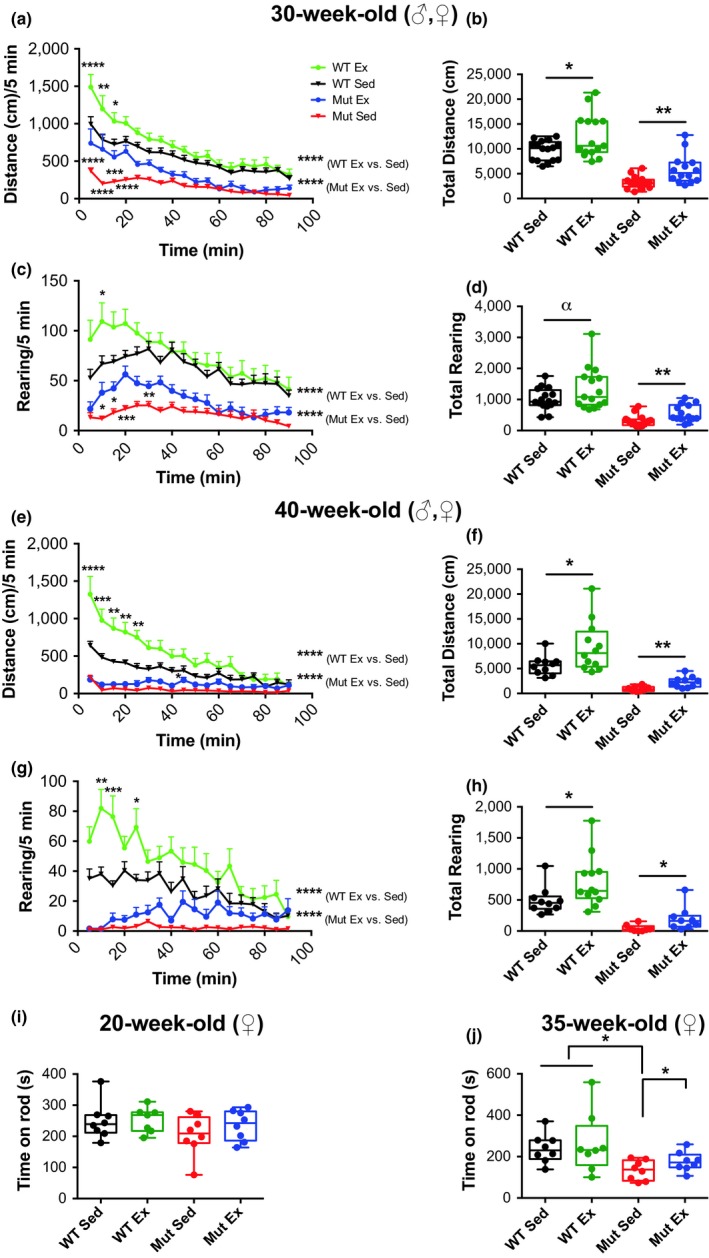
Effects of voluntary running on locomotion in WT and mtDNA mutator mice. (a–h) Spontaneous locomotor activity of 30‐ and 40‐week‐old male and female sedentary and exercising mtDNA mutator and WT mice. Both distance traveled (a, b) and rearing (c, d) over a 90‐min period were markedly increased by voluntary running in 30‐week‐old WT animals (WT Sed *N* = 16, WT Ex *N* = 15) and mtDNA mutator mice (Mut Sed *N* = 16, Mut Ex *N* = 14). (e–h) Notably, these improvements were sustained and even augmented at 40 weeks of age in both genotypes (WT Sed *N* = 10, WT Ex *N* = 12, Mut Sed *N* = 8, Mut Ex *N* = 10). (i, j) Exercising mtDNA mutator mice (females) performed better on the rotarod at 35 weeks of age than aged‐matched sedentary mice (*N* = 8 per group both time‐points). Significances were determined by two‐way *t* test (b, d, f, h–j) or two‐way ANOVA with post hoc analysis (a, c, e, g) with α *p* < .10, *p < .05, **p < .01, ***p < .001, and ****p < .0001

Spontaneous locomotor and rearing behaviors were monitored for 90 min in both males and females at 30 and 40 weeks of age. We found that total distance and rearing were both highest in WT exercising mice at both 30 and 40 weeks of age (Figure 2a–h, Figure S2). Hence, spontaneous exercise increased these locomotor parameters in WT mice. Sedentary mtDNA mutator mice had the lowest activity and rearing scores at both 30 and 40 weeks of age. Notably, exercise improved locomotion and rearing at both 30 and 40 weeks of age in mtDNA mutator mice, although the scores of exercised mtDNA mutator mice were always lower than those of WT mice with or without exercise (Figure 2a–h, Figure S2). For both WT and mtDNA mutator mice, we found that running and rearing activity decreased from 30 to 40 weeks of age in both sedentary and exercised groups (Figure 2a–h, Figure S2). When we tested forced motor coordination in females by rotarod performance at 20 weeks, we found that it was not significantly affected by either exercise or the PolG mutation, although there was a tendency for the sedentary mtDNA mutator mice to fall off the rotarod quicker as compared to the other three groups (Figure 2i). At 35 weeks of age, the time that mtDNA mutator female mice were able to stay on the rotarod was significantly shorter than that of WT mice. Notably, rotarod performance was significantly improved by exercise in female mtDNA mutator mice when tested at 35 weeks of age (Figure 2j).

### 2.4 Weight and lifespan

Exercise did not affect mtDNA mutator weight increase (Figure S3a and b), which leveled off at 20–30 weeks of age as expected (Trifunovic et al., 2004). Exercise did affect weight increase in WT female mice that appeared to cease gaining weight at 30 weeks as compared to WT sedentary mice (Figure S3a and b). Despite the dramatic effect of exercise on the mutator mouse phenotype, all seven voluntary exercising mtDNA mutator female mice died prematurely, and within the same 40 ± 5 week interval as the sedentary mtDNA mutator mice (Figure S3c). While our longevity test is underpowered for the detection of small or modest effects of exercise on longevity, it has been previously shown that sedentary mtDNA mutator mice never live > 100 weeks (Kujoth et al., 2005; Ross et al., 2013; Trifunovic et al., 2004). Thus, our data suggest that voluntary exercise is not causing any major increase in longevity.

### 2.5 Genotype, but not exercise, affects muscle histochemistry

**Figure 3:**
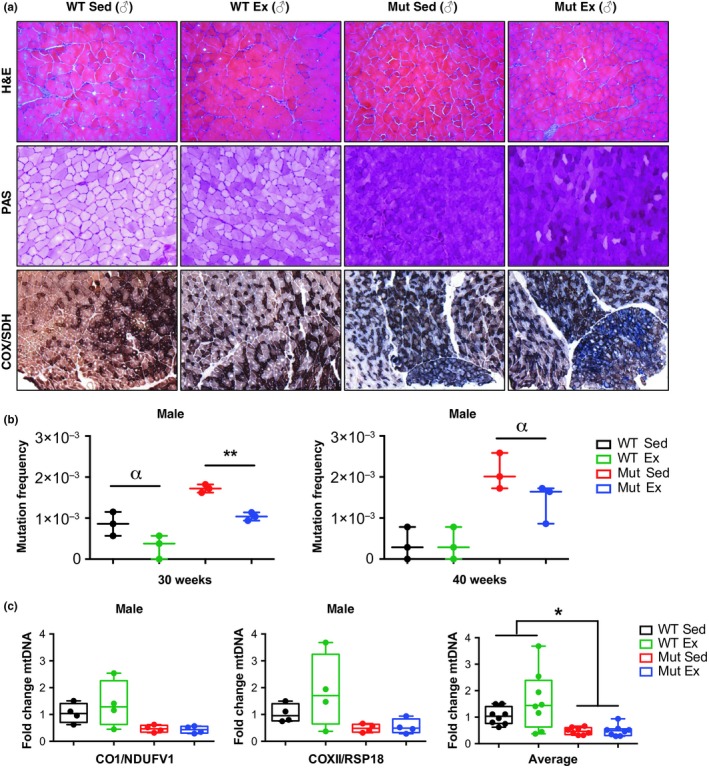
Effects of voluntary running on muscle histology, mitochondrial DNA mutations, and copy numbers. (a) Hematoxylin and eosin (H&E) stain for general tissue organization, periodic acid–Schiff (PAS) stain for glycogen, glycoproteins, and glycolipids, and cytochrome *c* oxidase/succinate dehydrogenase (COX/SDH) double‐enzyme histochemistry for mitochondrial function of gastrocnemius muscle from sedentary and exercising 30‐week‐old male WT and mtDNA mutator mice. Representative images were selected based on observation of muscle samples from 4 mice each from the 4 different conditions. (b) mtDNA mutation frequency comparisons from 30‐ and 40‐week‐old male gastrocnemius (*N* = 3 per condition) suggest that exercise decreases the total number of mutations in mtDNA mutator mice. (c) mtDNA copy number in 30‐week‐old gastrocnemius from male WT Ex, Mut Sed, and Mut Ex relative to WT Sed (*N* = 4 per condition) determined by qPCR with two different sets of primers shows no difference between Mut Sed and Mut Ex. Significances were determined by unpaired *t* test (b, c), with α *p* < .10, *p < .05, **p < .01

Histological analysis of the gastrocnemius muscle from 30‐week‐old males using H&E staining did not reveal major changes in the mutator mice comparing exercising and sedentary individuals, neither did PAS staining to evaluate levels of glycogen, glycoproteins and glycolipids, or COX*/*SDH dual‐labeling histochemistry (Ross, 2011) to monitor mitochondrial respiratory function. Unexpectedly, we found a strong increase in PAS staining of skeletal muscle tissue from both sedentary and exercised mtDNA mutator mice, compared to sedentary and exercised WT mice. This finding is most likely due to the fact that mtDNA mutator mice have increased glycogen storage although higher level of glycoproteins and proteoglycans cannot be excluded. The fact that muscle fibers appear somewhat heterogeneous in mtDNA mutator mice might suggest local metabolic changes in response to exercise (Jensen, Rustad, Kolnes, & Lai, 2011). Likewise, COX/SDH staining showed impaired mitochondrial respiratory function in skeletal muscle samples from both male sedentary and exercised mtDNA mutator mice, compared to WT mice (Figure 3a).

### 2.6 Exercise decreases mtDNA mutation load

At 30 and 40 weeks of age, the mtDNA mutation load was measured in male gastrocnemius muscle from all four groups by post‐PCR cloning (Figure 3b). As expected, sedentary mtDNA mutator mice had a higher mutation frequency (≈1.7 × 10^–3^ at 30 weeks of age and ≈2.1 × 10^–3^ at 40 weeks of age) as compared to WT mice (Kujoth et al., 2005; Ross et al., 2013; Safdar et al., 2011; Trifunovic et al., 2004). In accordance with a previous study that used forced endurance exercise (Safdar et al., 2011), we found that also voluntary exercise decreased the mutation frequency in mtDNA mutator mice, significantly so at 30 weeks (≈1.2 × 10^–3^; *p* < .05) when the mice were still actively running and nearly so at 40 weeks of age (≈1.4 × 10^–3^; *p* = .0677) when the mice had largely stopped to exercise. Notably, the mtDNA mutation frequency of 30‐week‐old exercising mutator mice was not significantly different from that of the sedentary WT mice used. It should be noted that the latter were derived during the breeding of heterozygous mtDNA mutator mice and that these “WT” mice therefore inherit a degree of mtDNA mutations from their heterozygous mother, with measurable effects on the offspring (Ross et al., 2013). When we measured the mtDNA copy number in 30‐week‐old male gastrocnemius tissue from all groups using quantitative PCR and targeting two different sets of mitochondrial and nuclear genes, we found that mutator mice have about 50% less mtDNA and that exercise did not affect mtDNA abundance (Figure 3c).

We have previously demonstrated that increasing or decreasing the mtDNA mutation load in mtDNA mutator offspring via the maternal germline can affect aging onset and progression as well as lifespan and incidence of brain malformations (Ross et al., 2013). Furthermore, we showed that these inherited mtDNA mutations can also alter aging onset, progression, and lifespan in "WT littermates" (PolG^wt/wt^), mice with a wild‐type nuclear DNA background (Ross, Coppotelli, Hoffer, & Olson, 2014). Our findings indicate that exercise can decrease the amount of mtDNA mutations in the prematurely aging mtDNA mutator mice without affecting the total amount of mtDNA. The fact that the total mtDNA copy number is low in mtDNA mutator mice and not affected by exercise is likely to increase the negative effects of the pathological mitochondria on the condition of the mtDNA mutator mice, as recently suggested (Jiang et al., 2017).

### 2.7 Protein dysregulation in mtDNA mutator mice is partially normalized by exercise in muscle and brain

**Figure 4:**
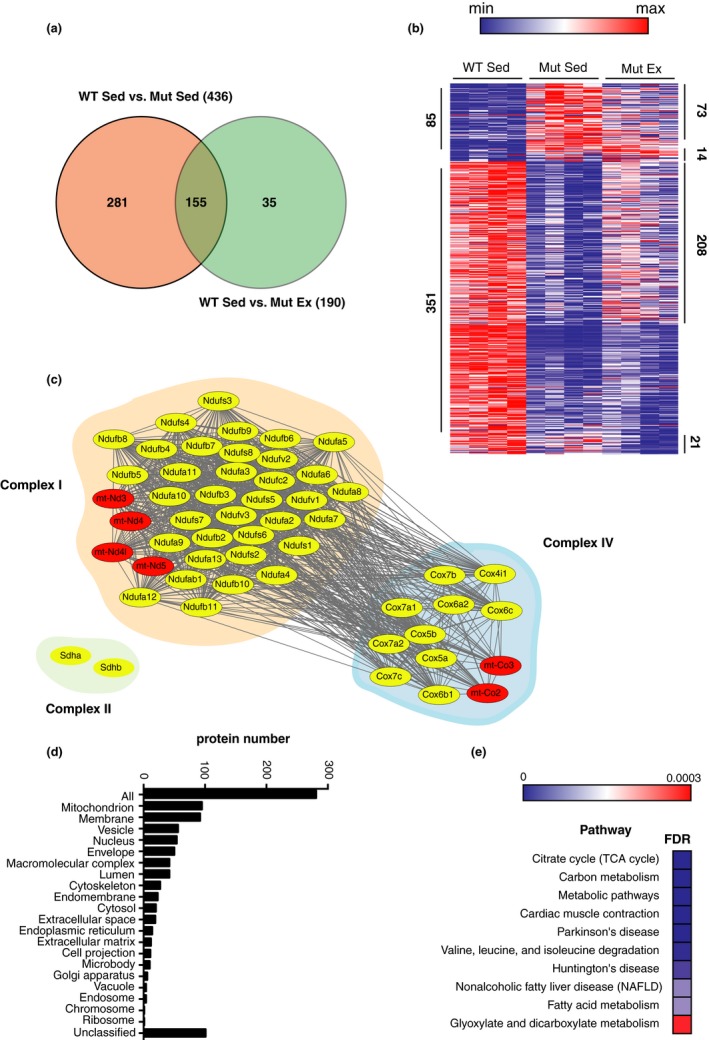
Effects of voluntary running on proteomic profiles in skeletal muscle. (a) Venn diagram depicting the total number of proteins differing between gastrocnemius from 30‐week‐old sedentary WT and mtDNA mutator mice (436) and exercising mtDNA mutator mice (190), with 281 deregulated proteins specific to mouse genotype, 35 proteins specific to the effect of exercise in mtDNA mutator mice, and 155 indicating the overlap between the two conditions (males, *N* = 4 each group). (b) Heat map representation of proteins found significantly deregulated in Mut Sed and Ex as compared to WT Sed. Eighty‐five proteins were found up‐regulated (red) and 351 down‐regulated (blue) when comparing sedentary WT and mtDNA mutator mice. The expression of 73 of the 85 up‐regulated proteins and 208 of the 351 down‐regulated proteins were normalized with exercise in mtDNA mutator mice. Interestingly, exercise induced up‐regulation of 14 proteins and down‐regulation of 21 proteins in mtDNA mutator mice. (c) STRING network analysis represented using the Cytoscape platform showing the specific subunits (red for mitochondrial‐encoded; yellow for nuclear‐encoded) of the OXPHOS complexes I and IV down‐regulated in the mutator mouse. (d) Gene Ontology and cellular component analysis of proteins normalized by exercise (281) in the mtDNA mutator mouse reveal about 100 proteins that belong to the mitochondrial compartment. (e) KEGG pathway enrichment analysis of normalized proteins in mtDNA mutator mice reveals the tricarboxylic acid (TCA) cycle as a top metabolic process improved by exercise in the mtDNA mice. FDR: false discovery rate

**Figure 5:**
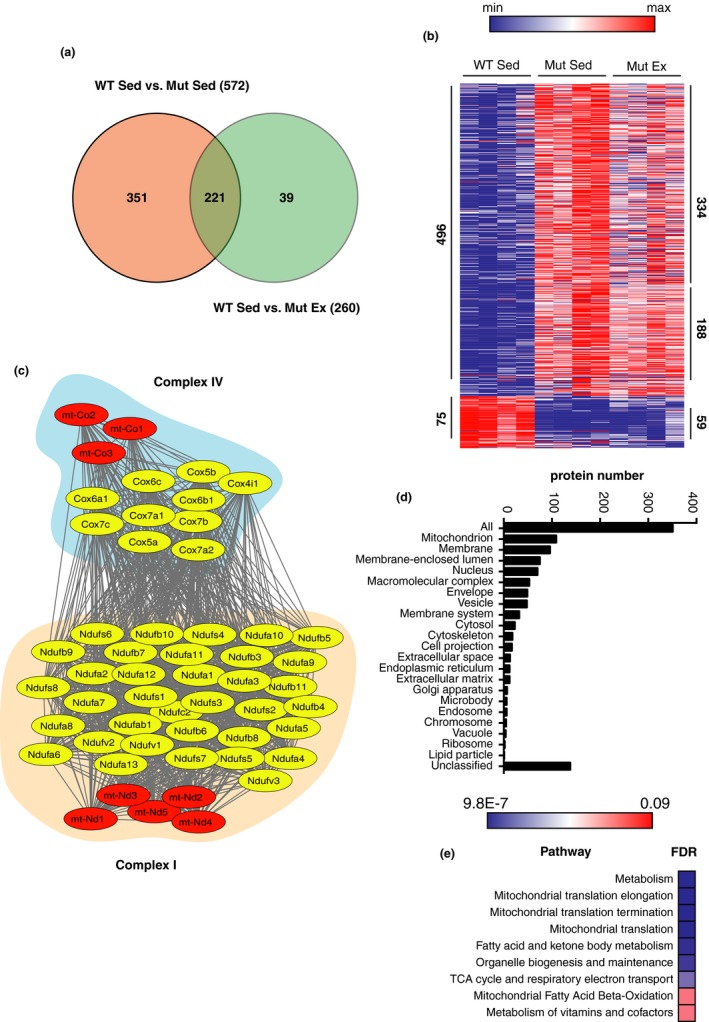
Effects of voluntary running on proteomic profiles in striatum. (a) Venn diagram illustrating the total number of altered proteins when comparing 30‐week‐old sedentary WT and mtDNA mutator mice (572), and exercising mtDNA mutator mice (260), with 351 proteins specific to mouse genotype, 39 proteins specific to the effect of exercise in mtDNA mutator mice, and 221 proteins present in both conditions (males, *N* = 4 each group). (b) Heat map representation of proteins found significantly deregulated in Mut Sed and Ex as compared to WT Sed. Proteomic analysis revealed 496 up‐regulated (red) and 76 down‐regulated (blue) proteins in sedentary mtDNA mutator mice, as compared to sedentary WT mice. Exercise normalized 334 of the 496 up‐regulated and 17 of the 76 down‐regulated proteins, but caused up‐regulation of 26 and down‐regulation of 12 other proteins. (c) STRING network analysis represented using the Cytoscape platform showing the specific subunits (red for mitochondrial‐encoded; yellow for nuclear‐encoded) of the OXPHOS complexes I and IV down‐regulated in mtDNA mutator mice. (d) Gene Ontology and cellular component analysis of proteins normalized by exercise (351) in the mtDNA mutator mouse reveal about 110 proteins that belong to the mitochondrial compartment. (e) KEGG pathway enrichment analysis of normalized proteins in mtDNA mutator mice reveals mitochondrial translation as a top biological process improved by exercise in the mtDNA mice. FDR: false discovery rate

Quantitative mass spectrometry revealed dramatic effects of having the mtDNA mutator genotype and of exercise on protein expression in all three tissues analyzed: gastrocnemius, striatum, and motor cortex, collected from 30‐week‐old male mice. When we compared sedentary WT and mutator mice, we found 436 and 572 differentially regulated proteins of ≈5.5 K and ≈10K in gastrocnemius and striatum, respectively, while only 47 proteins of ≈9.5 K detected in motor cortex were differentially regulated (Figures 4a and 5a, and Figure S4). Interestingly, the number of proteins identified in motor cortex was similar to the number of proteins identified in striatum, despite the fact that much fewer aberrant protein levels were found in motor cortex of mutator mice, suggesting that motor cortex was much less affected in mtDNA mutator mice than striatum. Comparing protein profiling between the three tissues analyzed, we find that numbers of proteins of relevance for membranes, and for mitochondria, ranked as the top two in all three tissues. Proteins of importance for the nucleus were ranked as 3 or 4 in the three investigated areas. This shows that basic alterations caused by impaired mitochondrial function are similar in cortical and subcortical brain areas, as well as in skeletal muscle. Alterations of other groups of proteins differed more between tissues. Remarkably, in gastrocnemius, the majority of the deregulated proteins were down‐regulated (351 of 436), while in striatum, the vast majority of deregulated proteins were up‐regulated (496 of 572) (Figures 4b and 5b). Nevertheless, the two tissues have 113 of the deregulated proteins in common, with the majority being down‐regulated (Figure S5a). Those down‐regulated proteins belong to the mitochondrial compartment and oxidative phosphorylation (OXPHOS) complexes, mainly complexes I and IV, as shown by Gene Ontology (GO), cellular component, and Kyoto Encyclopedia of Genes and Genomes (KEGG) pathway enrichment analyses (Figures 4c and 5c, Figure S5b). Analysis of the up‐regulated proteins in striatum revealed enrichment in several metabolic pathways, and in particular the valine, leucine, and isoleucine degradation pathways, suggesting that the tissue is diversifying substrate usage for energy production (Figure S5d). Several proteins involved in mtDNA synthesis and transcription (Polg, Polg2, Polrmt, Poldip2, Tfam, Tfem, Tfb1m, Tfb2m) and in mitochondrial translation (such as Mrps2, 6, 40, 31, 30, 22, 25, 16, 17, 9, 47, 34 etc.) were found to be up‐regulated in striatum of mutator mice, but not in skeletal muscle. While the proteins involved in mitochondrial quality control such as Fis1, Opa1, Lonp1, and Mfn1 were found down‐regulated in muscle, some were up‐regulated in striatum, such as Opa1 and Lonp1. This suggests better ability of striatal tissue to counteract mitochondrial dysfunction.

Notably, exercise mitigates the difference in protein expression in all three tissues analyzed. When we compare Mut Sed and Mut Ex to WT Sed mice, we found that exercise normalizes the expression of 281 proteins in gastrocnemius, 351 in striatum, and 25 in cortex (Figures 4a and 5a, Figure S4). Gene Ontology and cellular component analyses of proteins normalized by exercise in the mutator mice revealed enrichment of mitochondrial proteins and the TCA pathway in all tissues (Figures 4d and e, 5d and e, Figure S4d and e). Gene Ontology enrichment pathway analyses using the total list of identified proteins as background dataset were also performed and similar results were found when using the entire proteome as the background dataset (Figures S6 and S7). A deeper look at the OXPHOS and TCA cycle improvements showed that the nuclear‐encoded components of complex II, including SDH‐A and B, were down‐regulated in sedentary mutator muscle and that exercise normalized these levels in mtDNA mutator mice. In striatum, however, we found that the level of these subunits did not differ between sedentary WT and mutator mice; thus, no additional increases were found with exercise in mtDNA mutator mice. We did find, however, an up‐regulation in SDHAF2, a necessary scaffolding protein for complex II assembly, in both sedentary and exercising mutator mice, an alteration that was not found in muscle. Interestingly, in skeletal muscle, the proteins involved in mitochondrial quality controls such as Fis1, Opa1, Lonp1, and Mfn1 were among those normalized by exercise, suggesting an improvement in, and involvement of, mitochondrial quality control as one of the molecular mechanisms underlying the effect of exercise on muscle.

In‐depth proteomics also revealed significantly lower levels of intra‐mitochondrial coenzyme Q10A (COQ10A) in skeletal muscle in sedentary mtDNA mutator mice as compared to WT mice. This confirms what was previously found in heart from several mouse models of OXPHOS deficiency (Kühl et al., 2017). Interestingly, in striatum, several additional key biosynthesis enzymes (COQ2, COQ3, COQ4, COQ5, COQ6, COQ7) were up‐regulated, including COQ10A, thus again demonstrating tissue specificity in response to mitochondrial dysfunction. Moreover, no significant changes in COQ10A or COQ enzymes were detected in motor cortex from sedentary or exercising mtDNA mutator mice, which supports our hypothesis that cortex is more resistant to mitochondrial dysfunction than striatum. Exercise normalized dysregulated COQ10A protein levels in muscle and striatum, as well as several additional biosynthetic enzymes in striatum (COQ3, COQ5, COQ6, COQ7), thus suggesting that exercise and coenzyme Q supplementation could be used together to treat patients with OXPHOS dysfunction.

## 3 DISCUSSION

### 3.1 Use of the mtDNA mutator mouse for studies of exercise in aging

Exercise may be the single entity able to promote human health under essentially all circumstances (Reiner, Niermann, Jekauc, & Woll, 2013). The health trend of running includes an increasing aging population. The mtDNA mutator mouse (Trifunovic et al., 2004) demonstrates a very premature, but strikingly characteristic, number of aging phenotypes, including alopecia, graying of hair, loss of subcutaneous fat, kyphosis, osteoporosis, enlarged heart, anemia, impaired hearing, decreased fertility, sarcopenia, decreased body weight and size, and decreased lifespan, providing support for the mitochondrial hypothesis of aging, and a causative role of an increasing load of mtDNA mutations. We therefore tested the possible beneficial effects of voluntary running on aging of the mtDNA mutator mouse, with a particular focus on effects on protein homeostasis. We find that voluntary running ameliorates visible aging phenotypes and behavioral parameters of premature aging in agreement with a previous similar study, but does not appear to have a dramatic effect on longevity of mtDNA mutator female mice as previously demonstrated (Safdar et al., 2011). Notably, we used a regimen of voluntary exercise while forced exercise was used by Safdar et al. (Safdar et al., 2011), which perhaps could explain the different effect on longevity. The response to forced exercise has been compared to that of stress with the activation of the adrenal gland and sympathetic nervous system (Sasaki et al., 2016).

### 3.2 Observable improvements caused by running

Both male and female mtDNA mutator mice ran less than WT mice, and particularly so during the last 10 weeks of their 30 weeks of running period. Running length and exercise exhaustion might be correlated to processing certain metabolites, possibly mirrored by the increase in glycogen storage in both sedentary and exercising mtDNA mutator (male) mice (Figure 3a). Nevertheless, we find that visual characteristics such as alopecia, graying of the hair, body size reduction, and kyphosis were markedly reduced in both sexes by voluntary running of mtDNA mutator mice as observed at 35 weeks of age, when these mice had been running from 10 weeks of age. Similarly, running improved open‐field locomotion and rearing of both male and female mtDNA mutator mice, although their performance in these tests remained well below those of WT mice. We noted that exercise also increased locomotion and rearing activity of WT mice, suggesting that the restricted laboratory cage environment is insufficient for the development of the full locomotion potential of WT mice. Finally, rotarod tests in WT female mice were not affected by exercise, while mtDNA mutator female mice at 35 weeks of age performed better if they had exercised. However, rotarod performance was relatively less impaired in both sedentary and exercising mtDNA mutator mice than open‐field activity and rearing. This demonstrates the ability to perform better in a forced rather than in a voluntary behavior test, as can also be seen in the MitoPark mouse model for Parkinson's disease (Chen et al., 2019). Together, the visual and behavioral improvements of health demonstrate that exercise can partially counteract the effects of a severe, pan‐cellular increase of mtDNA mutations.

### 3.3 No major improvement of lifespan by voluntary running

Unexpectedly, the lifespan of mtDNA mutator mice did not appear to be significantly affected by voluntary exercise. All seven females in the exercised mtDNA mutator group died as prematurely as the seven sedentary mutator mice, within 40 ± 5 weeks of age. However, due to the size of the cohorts studied, we cannot preclude that voluntary exercise might have had a small or modest effect on longevity. In addition to the possibility that voluntary exercise does not induce the same stress effects as forced exercise, another explanation may be the inability of voluntary running to improve stem cell populations, which have been reported to be significantly defective in mtDNA mutator mice (Ahlqvist et al., 2012; Fox, Magness, Kujoth, Prolla, & Maeda, 2012). Understanding how different forms of exercise affect lifespan extension should be further investigated since forced endurance exercise has reportedly improved longevity in this mouse model (Safdar et al., 2011).

### 3.4 Exercise affects mtDNA mutation load more than mtDNA copy number

The mtDNA mutation load in gastrocnemius muscle was found to be several folds higher in mtDNA mutator (male) mice than in WT mice, in accordance with previous studies (Kujoth et al., 2005; Ross et al., 2013; Safdar et al., 2011; Trifunovic et al., 2004). This load was markedly reduced in 30‐week‐old mtDNA mutator mice that were still actively running, in accordance with a previous study that showed that forced exercise can ameliorate aging phenotypes in a similar mouse model (Safdar et al., 2011). However, mtDNA mutation levels were still above those of sedentary or exercised WT mice, showing that the exercising mutator mice were still burdened by pathological levels of mutated mtDNA. At 40 weeks of age, when mtDNA mutator mice had largely ceased running, an exercise‐induced decrease of mtDNA mutation load was no longer significant (*p* = .0677). These data thus demonstrate that voluntary running improves several premature aging phenotypes in the mutator mouse, including mtDNA mutation load. However, our findings do not suggest that the decrease in mutations per se is responsible for mediating the benefits of exercise. We also found that the mtDNA copy number in 30‐week‐old male mtDNA mutator mice was less than 50% of that of WT mice. This is lower than the ~70% of control levels originally found in mtDNA mutator mice (Trifunovic et al., 2004), possibly due to anticipation effects across generations (Ross et al., 2013). While a clear tendency for running to increase mtDNA copy number was seen in WT mice, this was not the case in mtDNA mutator mice. This suggests that quality control mechanisms, presumably including mitophagy of malfunctioning mitochondria, keep the mtDNA copy number low in both sedentary and exercising mtDNA mutator mice. Low mtDNA copy number per se has been linked to general illness and disease pathology, including Parkinson's disease (Pyle et al., 2016), oxidant‐induced cell damage (Huang et al., 2016), and cancers (Memon et al., 2017). Furthermore, there is evidence to suggest that a sufficient number of mtDNA molecules per se may be able to improve certain pathologies regardless of mtDNA mutation load (Jiang et al., 2017).

The “WT” mice used in this study are PolgA^wt/wt^ littermates obtained when using PolgA^wt/mut^ heterozygous parents to generate PolgA^mut/mut^ homozygous mtDNA mutator mice. Since the mother is heterozygous, the mitochondria provided by her to the “WT” mice will carry a modest amount of mtDNA mutations, but there will be no further enhanced increase since the “WT” lacks mutated PolgA (Ross et al., 2013). We find a tendency for running to have decreased the mtDNA mutation load in 30‐week‐old WT mice. This suggests that effects of exercise on mtDNA mutation load can be seen in mice with much lower mutation levels than those in mtDNA mutator mice. Moreover, we found that mtDNA copy number was improved by exercise in these “WT mice,” suggesting that either a different mechanism, such as mitophagy is affecting copy number, or that the number of mtDNA molecules cannot actually be increased in mtDNA mutator mice.

### 3.5 Exercise effects at the proteomic level

We note that proteins were dysregulated in all three areas studied (gastrocnemius, striatum, and cortex cerebri) in 30‐week‐old male mtDNA mutator mice and that exercise normalized many of these dysregulations in all three regions. Importantly, we found that mtDNA mutator mice allowed to exercise had an increase of proteins involved in the TCA with no actual improvement in OXPHOS complex proteins except for complex II, the proteins of which are entirely encoded by nDNA. Thus, there seems to be an effort of the system to ramp up OXPHOS, but only nDNA‐dependent systems (TCA and complex II) could become improved. In line with these observations, the COX/SDH histochemistry of muscle tissue shows that skeletal muscle fibers remain OXPHOS deficient in exercising mtDNA mutator mice.

In gastrocnemius muscle, ~80% of the dysregulated proteins were down‐regulated, while in striatum, ~87% of the dysregulated proteins were instead up‐regulated. Among the up‐regulated protein groups in mtDNA mutator striatum, were those involved in the use of substrate molecules for energy production, mtDNA synthesis and transcription, and translation. There was down‐regulation of some of the proteins involved in mitochondrial quality control and coenzyme Q biosynthesis in gastrocnemius, and up‐regulation of some such proteins in striatum, suggesting active coping mechanisms in response to impaired mitochondrial function. Moreover, our analyses revealed that the cerebral cortex proteome is much less affected than gastrocnemius muscle and striatum proteomes, suggesting higher robustness or increased protection of this brain region. Recent studies demonstrate that striatum is more sensitive to changes in OXPHOS, Ca^2+^ buffering capacity, and mtDNA quality (Brustovetsky et al., 2003; Oliveira & Gonçalves, 2009; Pickrell, Pinto, Hida, & Moraes, 2011), supporting this hypothesis. Together, our findings demonstrate that the brain has a better capability to cope with mitochondrial dysfunction, and motor cortex better so than striatum, as compared to skeletal muscle, perhaps consistent with the constantly high‐energy demand in the brain.

We conclude that voluntary exercise has profound positive effects on appearance, behavior, mtDNA mutations, and the proteome in prematurely aging mtDNA mutator mice, although normal conditions are not reached and longevity does not appear to be improved. It is thus suggested that the positive effects of exercise in aging humans also involve decrease of the mtDNA mutation load and normalization of neural and skeletal muscle protein levels, especially those involved in the TCA cycle and coenzyme Q.

## 4 EXPERIMENTAL PROCEDURES

### 4.1 Animals

Homozygous mtDNA mutator (*PolgA* ^mut/mut^) mice (*N* = 8 males, *N* = 8 females) and “wild‐type” (*PolgA* ^wt/wt^) nDNA^wt^/mtDNA^mut^ littermates (*N* = 8 males, *N* = 8 females) that have a wild‐type nuclear genome but maternally inherited mtDNA mutations (Ross et al., 2014, 2013), herein referred to as WT mice, were obtained by crossing mice heterozygous (*PolgA* ^WT/mut^) for the mtDNA mutator allele. Animals were genotyped as previously described (Trifunovic et al., 2004). All mice had received a standard diet (R34, Lactamin/Lantmännen, Stockholm, Sweden) and water ad libitum, were kept on a 12:12 hr light:dark cycle at 22–23°C, and had access to a small house and tissues for nesting. Adequate measures were taken to minimize pain and discomfort. Investigation has been conducted in accordance with the ethical standards and according to the Declaration of Helsinki and national and international guidelines and has been approved by the authors’ institutional review board.

### 4.2 Running wheels

Mice were grouped based on sex and genotype, with 2 mice of same sex and genotype per cage, for the duration of the experiment. A total of 16 mice per genotype and condition with equal numbers of males and females were used (Figure 1a). Mice in the exercise group had free access to running wheels in home cages (1 wheel per cage). A running wheel system (Columbus Instruments, Columbus, OH, USA) compatible with standard mouse home cages was used, thus allowing for long‐term studies and preservation of social interaction. Number of wheel rotations, duration, and time of day were recorded.

### 4.3 Locomotion and rearing

Spontaneous locomotor activity in an open field was studied in male and female sedentary and exercising mtDNA mutator mice and WT littermates at 30 weeks of age (Males: WT Sed *N* = 8, WT Ex *N* = 7, Mut Sed *N* = 8, Mut Ex *N* = 6; Females: WT Sed *N* = 8, WT Ex *N* = 8, Mut Sed *N* = 8, Mut Ex *N* = 8) and 40 weeks of age (Males: WT Sed *N* = 4, WT Ex *N* = 4, Mut Sed *N* = 3, Mut Ex *N* = 3; Females: WT Sed *N* = 6, WT Ex *N* = 8, Mut Sed *N* = 5, Mut Ex *N* = 7). A multi‐cage infrared‐sensitive motion detection system (AccuScan Instruments, Columbus, OH, USA) was used to record spontaneous locomotion. Total distance and rearing “rearing were recorded” recorded in 5‐min intervals over 90 min. Total distance traveled (horizontal activity) was recorded in centimeters, and rearing (vertical activity) was determined by sensor beam‐breaks using appropriate software (Versamax Activity Monitor, AccuScan Instruments). Transparent locomotor chambers (40 × 40 × 30 cm) were equipped with a grid of infrared beams at floor level and 7.5 cm above. Wooden shavings were placed on the floor of the boxes prior to testing. Animals were habituated in a dimly lit, low‐noise, and ventilated experimental room kept at 20–22°C for 1 hr prior to testing. Experiments were performed between 12.00 and 17.00 (light phase), and the locomotor boxes were cleaned with 70% ethanol after each test period.

### 4.4 Rotarod Performance

Female sedentary and exercising mtDNA mutator mice and WT littermates (*N* = 8 per group) were tested at 20 weeks and 35 weeks of age. Subjects in their home cages were placed in a testing room for at least 1 hr. After acclimation, mice were placed on the rod three times for 1 min at 5 rpm with 10‐min inter‐trial intervals to become accustomed to the rotarod. Mice were then returned to the home cages for 1 hr before testing. Three trials with 30‐min intervals were recorded during a testing session. The apparatus was set to accelerate from 4 to 40 rpm in 300 s, and animals from the same cage were placed in separate lanes on the rod. A trial begins when accelerating rotation starts and ends when an animal falls off the rod. Latency to fall was recorded.

### 4.5 Phenotype scoring

The presence of alopecia, graying of the hair, body size reduction, and kyphosis was scored in 35‐week‐old male and female sedentary and exercising mtDNA mutator and WT mice (*N* = 3 per group) as previously described (Ross et al., 2013) using a scale of 0–3 (0, absence of phenotype; 1, moderate presence of phenotype; 2, strong presence of phenotype; and 3, severe presence of phenotype) for each of the four symptom types and summed such that 12 would represent the most severe aging score. Mice were scored with identity blinded, thus without knowledge of genotype or whether the scored mouse had exercised or not.

### 4.6 Tissue preparation for cryosectioning

For preparation of fresh‐frozen sections for histochemistry, male sedentary and exercising mtDNA mutator and WT animals were killed by cervical dislocation. Gastrocnemius tissues were rapidly frozen on dry ice and stored at −80°C. Frozen tissues were embedded (Tissue‐Tek; Sakura Finetek), and 14‐μm cryostat (Microm Model HM 500M Cryostat; Microm) sections were taken at −21°C, thawed onto slides (Super Frost; Menzel‐Gläser), and stored at −20°C until use.

### 4.7 Histology and enzyme histochemistry

For visualization of skeletal muscle morphology, gastrocnemius sections from 30‐week‐old male sedentary and exercising mtDNA mutator mice and WT littermates (*N* = 4 per group) were stained with hematoxylin and eosin (H&E), dehydrated in increasing concentrations of ethanol (70%, 95%, and 99.5%), mounted (Entellan, VWR International AB), and coverslipped. Periodic acid–Schiff (PAS) staining, used to show glycogen, glycoproteins, and glycolipids, was performed by immersing the slides in periodic acid solution for 5 min at room temperature. After washing with water, slides were immersed in Schiff's reagent for 15 min at room temperature (18–26°C). Following rinsing for 5 min in running water, sections were dehydrated in increasing concentrations of ethanol (70%, 95%, and 99.5%), mounted with Entellan, and coverslipped. To visualize respiratory dysfunction, we used enzyme histochemistry to determine the activities of cytochrome *c* oxidase (COX) and succinate dehydrogenase (SDH) as previously described (Ross, 2011).

### 4.8 Microscopy

Brightfield microscopy was used to evaluate and document histological results (Zeiss Axiophot 2 and Axioplan 2 Imaging Software, Zeiss Axiophot 2; Carl Zeiss).

### 4.9 mtDNA mutation load analysis

For the DNA extraction, gastrocnemius tissues from three male mice per group were lysed in buffer containing 50 mm Tris–HCl, pH 8.5, 1 mM EDTA, 0.5% Tween‐20, and 200 ng/ml proteinase K at 55°C for 6 to 12 hr with gentle shaking (400–600 rpm). Lysates were vortexed vigorously, and the nonsoluble fractions were pelleted by centrifugation (8,000 g for 15 min). After transferring the supernatant to a new 2‐ml Eppendorf tube, phenol/chloroform/isoamyl alcohol (25:4:1), solution was added. Samples were mixed vigorously and centrifuged (8,000 g for 15 min), after which 0.45–0.5 ml of the supernatant was transferred to a new 2‐ml tube. An equal volume of chloroform was added to the supernatant, and after vigorous mixing, the tube was centrifuged (8,000 g for 15 min). A total of 0.4 ml of the resulting supernatant was transferred to a new tube and mixed with 0.04 ml NaAc (3 M) and 0.44 ml isopropanol. The tube was maintained at −20°C for 10 min to facilitate DNA precipitation and then centrifuged (8,000 g for 15 min) to pellet the DNA. After discarding the supernatant, the DNA pellets were washed with 1 ml 70% ethanol, air‐dried, and dissolved in 0.4 ml Tris–EDTA (TE) buffer. A total of 500 ng DNA was used as a template to amplify a 462 bp fragment of the cytochrome *b* gene (primers Fw: AAA*AAGCTT*attccatttcacccctacta and Rev: AAA*GGATCC*aattcctgagattggtataa) with a high fidelity Pfu DNA polymerase (Promega, Madison, WI, USA). The PCR products were subjected to electrophoresis on a 1.5% agarose gel, and the DNA was purified (QIAquick Gel Extraction Kit Qiagen). After purification, the fragments were cloned in a pUC19 vector at the HindIII/BamHI restriction sites. Recombinant plasmids were identified by blue–white color selection. White colonies were grown in LB for 8 hr, and the DNA was extracted and sequenced by Sanger sequencing. Twenty‐four colonies were analyzed for each condition (8 colonies per mouse per condition) giving about 10,000 reads for each condition. Sequences were analyzed with DNA Dynamo Sequence Analysis Software, and mutation frequency was calculated by dividing the number of mutated bases by the number of bases sequenced.

### 4.10 mtDNA copy number

For relative mtDNA copy number determination, total DNA extractions were prepared as described above. Four biological replicates derived from 30‐week‐old male gastrocnemius for each condition were used, and two reactions with two different sets of primers were run: mtCO2/RSP18 and mtCO1/NDUFV1. Primer sequences were as follows: mtCO2 fw‐ATAACCGAGTCGTTCTGCCAAT and rev‐TTTCAGAGCATTGGCCATAGAA, RSP18 fw‐TGTGTTAGGGGACTGGTGGACA and rev‐CATCACCCACTTACCCCCAAAA, mtCO1 fw‐TGCTAGCCGCAGGCATTAC and rev‐GGGTGCCCAAAGAATCAGAAC, and NDUFV1 fw‐CTTCCCCACTGGCCTCAAG and rev‐CCAAAACCCAGTGATCCAGC. Quantification of mtDNA levels was done using SYBR‐Green qPCR analyses, and all data were normalized against wild‐type levels.

### 4.11 Tissue lysis, protein extraction, digestion, and peptide labeling with TMT10plex

Motor cortical and striatal brain regions and gastrocnemius were collected from 30‐week‐old male sedentary and exercising mtDNA mutator and WT animals (*N* = 4 per group). Tissues were lysed in SDS‐lysis buffer (4% (w/v) SDS, 25 mM HEPES pH 7.6, 1 mM DTT). Lysates were then heated at 95°C for 5 min in a thermomixer and sonicated with a sonicator probe to shear DNA. Samples were centrifuged at 14,000 g to remove cell debris, the supernatants collected and protein concentration estimated by the DC protein assay (Bio‐Rad). From each sample, 250 µg of total protein was taken and processed according to the FASP (Filter aided sample preparation) protocol (Wiśniewski, Zougman, Nagaraj, & Mann, 2009) with one modification: samples were digested on the filter with Lys‐C for 3 hr prior to trypsin digestion (16 hr). Peptide concentration was estimated by the DC protein assay (Bio‐Rad), and 100 µg of peptides from each sample were labeled with TMT10plex (Thermo Fisher Scientific) according to the manufacturer's instructions. See Table S1 for TMT sample arrays.

### 4.12 High‐resolution isoelectric focusing (HiRIEF) separation

After pooling the samples that belong together in each TMT set, each TMT set was cleaned by strong cation exchange solid phase extraction (SCX‐SPE, Phenomenex Strata‐X‐C, P/N 8B‐S029‐TAK). After drying in a SpeedVac (Thermo SPD111V with refrigerated vapor trap RVT400), the equivalent to 400 µg of peptides of each TMT‐pooled sample was dissolved in 250 µl of 8 M urea, 1% pharmalyte (broad range pH 3–10, GE Healthcare, P/N 17‐0456‐01), and this solution was used to rehydrate the IPG drystrip (pH 3–10, 24 cm, GE Healthcare, P/N 17‐6002–44) overnight. Focusing was done on an Ettan IPGphor 3 system (GE Healthcare) ramping up the voltage to 500 V for 1 hr, then to 2,000V for 2 hr, and finally to 8,000 V for 6 hr, after which voltage was held at 8,000 V for additional 20 hr or until 150 kVh was reached. After focusing was complete, a well‐former with 72 wells was applied onto each strip, and liquid‐handling robotics (Ettan Digester from GE Healthcare, which is modified from a Gilson liquid handler 215), using three rounds of different solvents ((a) milliQ water, (b) 35% acetonitrile, and (c) 35% acetonitrile, 0.1% formic acid), added 50 µl of solvent to each well and transferred the 72 fractions into a microtiter plate (96 wells, PP, V‐bottom, Greiner P/N 651201), which was then dried in a SpeedVac (Thermo SPD111V with refrigerated vapor trap RVT400).

### 4.13 Liquid chromatography–mass spectrometry (LC‐MS) analysis

For each LC‐MS run of a HiRIEF fraction, the autosampler (Ultimate 3,000 RSLC system, Thermo Scientific Dionex) dispensed 15 µl of mobile phase A (97% water, 3% acetonitrile, 0.1% formic acid) into the corresponding well of the microtiter plate (96 wells, PP, V‐bottom, Greiner P/N 651201), mixed by aspirating/dispensing 10 µl ten times, and finally injected 7 µl into a C18 guard desalting column (Acclaim PepMap 100, 75 µm × 2 cm, nanoViper, Thermo). After 5 min of flow at 5 µl/min with the loading pump, the 10‐port valve switched to analysis mode in which the NC pump provided a flow of 250 nl/min through the guard column. The curved gradient (curve 6 in the Chromeleon software) then proceeded from 3% mobile phase B (95% acetonitrile, 5% water, 0.1% formic acid) to 45%B in 45 min followed by wash at 99%B and re‐equilibration with initial conditions. Total LC‐MS run time was 69 min. We used a nano EASY‐Spray column (PepMap RSLC, C18, 2 µm bead size, 100Å, 75 µm internal diameter, 50 cm long, Thermo) on the nanoelectrospray ionization (NSI) EASY‐Spray source (Thermo) at 60°C. Online LC‐MS was performed using a hybrid Q‐Exactive mass spectrometer (Thermo Scientific). FTMS master scans with 70,000 resolution (and mass range 300–1600 m/z) were followed by data‐dependent MS/MS (35,000 resolution) on the top 5 ions using higher energy collision dissociation (HCD) at 30% normalized collision energy. Precursors were isolated with a 2 m/z window. Automatic gain control (AGC) targets were 1e6 for MS1 and 1e5 for MS2. Maximum injection times were 100ms for MS1 and 150 ms for MS2. Dynamic exclusion was used with 30 s duration. Precursors with unassigned charge state or charge state 1 were excluded. An underfill ratio of 1% was used.

### 4.14 Proteomics database search

All MS/MS spectra were searched by MSGF+/Percolator under the Galaxy platform (https://usegalaxy.org) using a target‐decoy strategy. The reference database used was the UniProt reference *Mus musculus* proteome (canonical and isoform, 51,529 protein entries, downloaded from uniprot.org on 2014‐08‐01). We used a precursor mass tolerance of 10 ppm and high‐resolution setting on MS2 level. Only peptides with fully tryptic termini were allowed. We considered carbamidomethylation on cysteine and TMT‐10plex on lysine and N‐terminus as fixed modifications, and oxidation of methionine as variable modification. Quantification of TMT‐10plex reporter ions was done using an integration window tolerance of 10 ppm. PSMs and peptides were filtered at 1% FDR (peptide level), and proteins were filtered additionally at 1% FDR (protein level) using the “picked” protein FDR method (Savitski, Wilhelm, Hahne, Kuster, & Bantscheff, 2015). The mass spectrometry proteomics data have been deposited to the ProteomeXchange Consortium via the PRIDE partner repository with the dataset identifier PXD011741.

### 4.15 Statistical analyses using SAM

To compare protein levels between conditions (sedentary vs. exercise in WT and mtDNA mutator mice), Student's *t* tests were applied on log2‐transformed data using SAM (Significance Analysis of Microarrays, http://statweb.stanford.edu/~tibs/SAM/) under R (version 3.2.2, The R Foundation for Statistical Computing). SAM performs *t* tests using permutation‐based corrections for multiple comparisons. Although originally designed for array data, SAM has been shown to be valid also for LC‐MS/MS data (Bereczki et al., 2018; Roxas & Li, 2008; Sandberg et al., 2012).

### 4.16 Gene Ontology and pathway enrichment analyses

Significantly de‐regulated proteins (FDR ≤ 0.05) were analyzed with the freely accessible WEB‐based software analysis tool GEne SeT AnaLysis Toolkit (WebGestalt) (http://www.webgestalt.org/option.php) (Wang, Duncan, Shi, & Zhang, 2013). Enrichment for biological processes, molecular function, and cellular component was performed by Gene Ontology (GO) analysis (Ashburner et al., 2000; The Gene Ontology Consortium, 2019). Enriched pathway analysis was performed with Kyoto Encyclopedia of Genes and Genomes (KEGG) pathway enrichment analysis. Functional protein–protein interaction was determined using the STRING database (Szklarczyk et al., 2015) and visualized using Cytoscape (Shannon et al., 2003).

### 4.17 Statistical analysis

Data are presented to 3 significant digits as number of observations, percentages, or mean values (M) with either *SEM* or minimum and maximum of the data set (boxplot). Statistical analyses, unpaired *t* test, two‐way ANOVA with post hoc analysis, and log‐rank Mantel–Cox, were performed with an alpha level of 0.05 using appropriate software (GraphPad Prism v. 6, GraphPad Software Inc, San Diego, CA, USA). Significances are denoted as *p < .05, **p < .01, ***p < .001, and ****p < .0001, with α *p* < .10 indicating a trend.

## CONFLICTS OF INTEREST

The authors declare no conflict of interest in relation to this work. David Sinclair: DAS is founder, equity owner, board member, advisor, director, consultant, investor and/or inventor of patents licensed to Vium, Jupiter Orphan Therapeutics, Cohbar, Galilei Biosciences, GlaxoSmithKline, OvaScience, EMD Millipore, Wellomics, Inside Tracker, Caudalie, Bayer Crop Science, Longwood Fund, Zymo Research, EdenRoc Sciences with affiliates, and Life Biosciences with affiliates. DAS is an inventor on a patent application filed by Mayo Clinic and Harvard Medical School and licensed to Elysium Health; his personal royalty share is directed to the Sinclair lab. For more information see: https://genetics.med.harvard.edu/sinclair-test/people/sinclair-other.php

## AUTHOR CONTRIBUTIONS

JMR, GC, and LO conceived the project and designed the research; JMR, GC, RMB, and KMK performed research; JL and DAS contributed analytic tools; JMR, GC, and RMB analyzed data; and JMR, GC, and LO wrote the paper.
