# Supplementary material for: Voluntary exercise normalizes the proteomic landscape in muscle and brain and improves the phenotype of progeroid mice
Source: Aging Cell. 2019 Sep 6;18(6):e13029. doi: 10.1111/acel.13029 (PMC6826127; doi:10.1111/acel.13029)
Supplement: Supplementary file 1 [file ACEL-18-e13029-s001.pdf]

Supplementary Information for

Voluntary exercise normalizes the proteomic landscape in muscle and brain and improves the phenotype of progeroid mice

Jaime M. Ross, Giuseppe Coppotelli, Rui M. Branca, Kyung M. Kim, Janne Lehtiö, David A. Sinclair, Lars Olson

Jaime M. Ross, Email: Jaime.Ross@ki.se or Jaime_Ross@hms.harvard.edu
Giuseppe Coppotelli; Email: Giuseppe_Coppotelli@hms.harvard.edu

This PDF file includes:

Figs. S1 to S7
Table S1

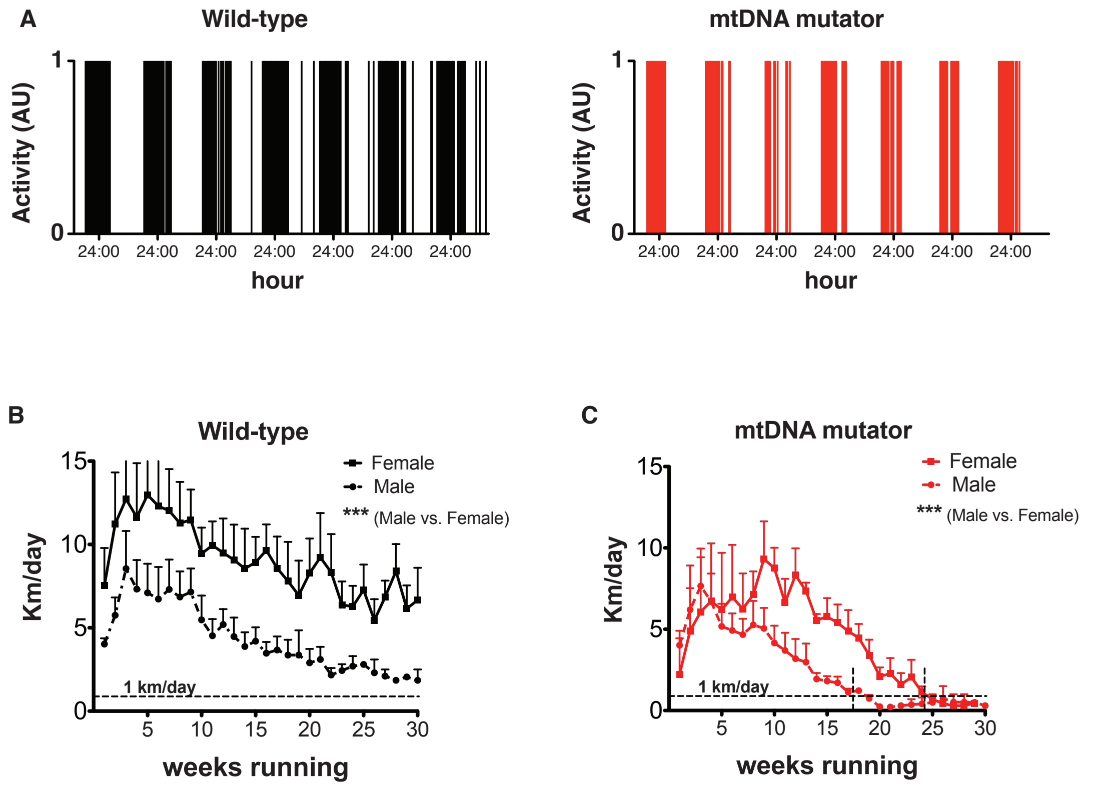

Fig. S1. Circadian activity rhythm and voluntary running of males and females. (A) Diurnal running wheel activity pattern. The circadian rhythm is strictly maintained in mtDNA mutator mice (room lights are on a 12:12 cycle), with "0" for no wheel movement and "1" indicating wheel movement. (B, C) Running activity in WT (N=8 male, N=8 female) and mtDNA mutator (N=8 male, N=8 female) mice comparing males and females in the same graph. Figure 1 shows these data comparing WT and mtDNA mutator mice in the same graph. Significances were determined by two-way ANOVA with posthoc analysis, with ***P<0.001.

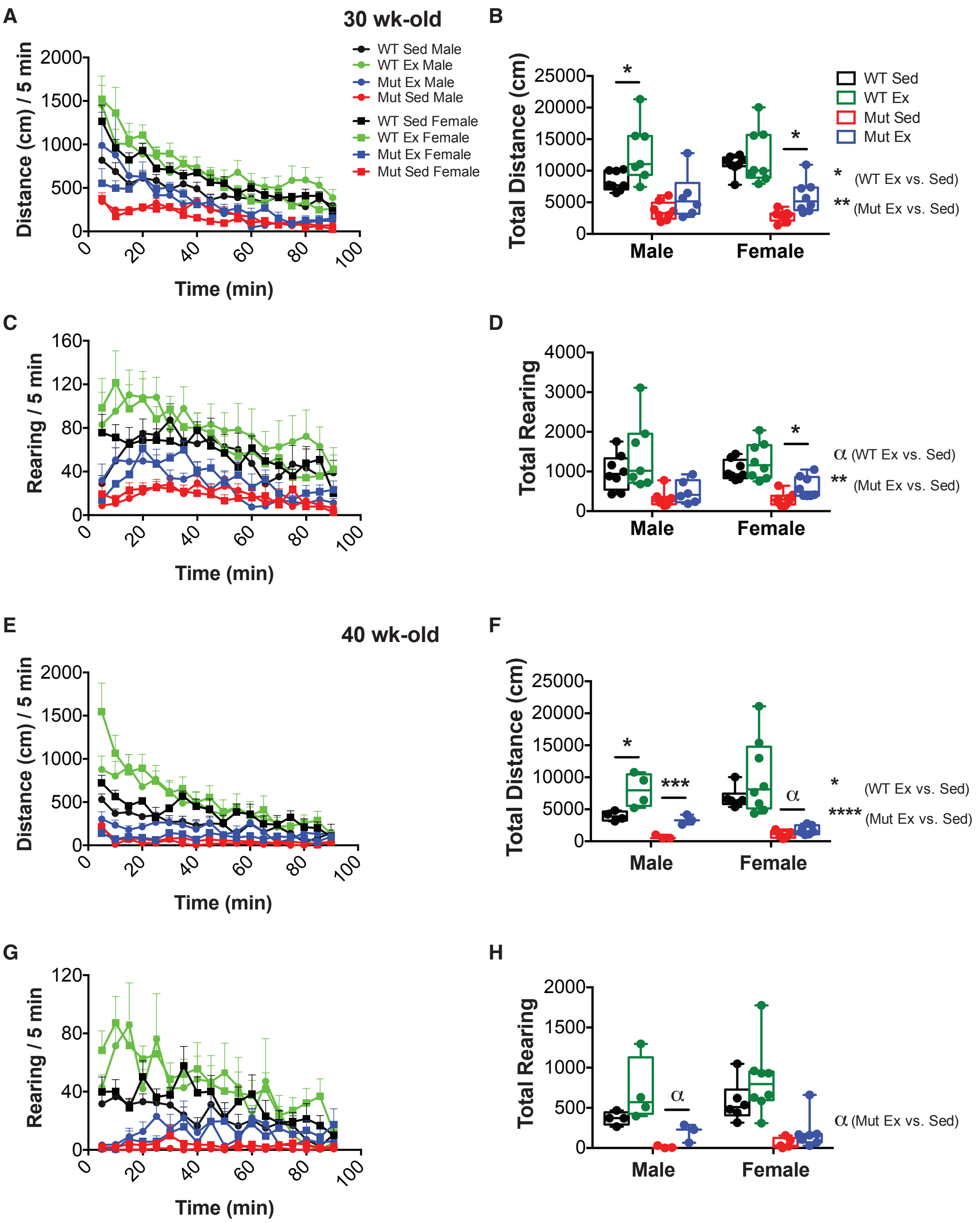

Fig. S2. Effects of exercise on locomotion in males and females. (A-H) Spontaneous locomotor activity of male and female sedentary and exercising mtDNA mutator and WT mice at 30 weeks of age (Males: WT Sed N=8, WT Ex N=7, Mut Sed N=8, Mut Ex N=6; Females: WT Sed N=8, WT Ex N=8, Mut Sed N=8, Mut Ex N=8) and 40 weeks of age (Males: WT Sed N=4, WT Ex N=4, Mut Sed N=3, Mut Ex N=3; Females: WT Sed N=6, WT Ex N=8, Mut Sed N=5, Mut Ex N=7). (A-D) After 10 weeks of voluntary running, both distance traveled (A, B) and rearing (C, D) were improved in both 30 week-old male and female mtDNA mutator and WT mice. (E-H) After 20 weeks of exercise, many of these increases were maintained and continued to improve in 40 week-old males and females of both genotypes. Significances were determined by two-way ANOVA with posthoc analysis, with α $P<0.10$, *$P<0.05$, **$P<0.01$, ***$P<0.001$, and ****$P<0.0001$.

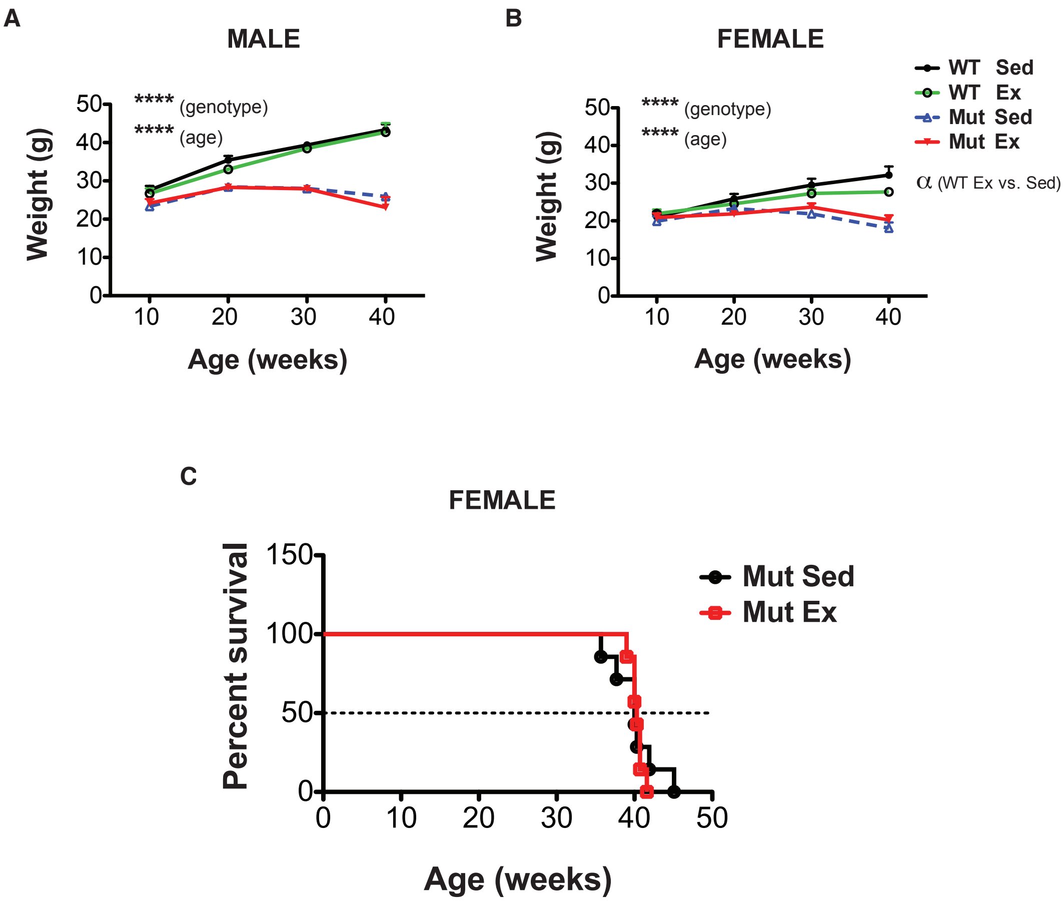

Fig. S3. Effects of voluntary running on body weight and lifespan. (A, B) Longitudinal body weights collected in sedentary and exercising WT and mtDNA mutator mice (N=8 per group except: 40 week-old males N=4 per group, 40 week-old females Mut Sed N=6, and 40 week-old females Mut Ex N=7) indicate minimal changes in body weight with exercise, with females slightly more affected than males. (C) Kaplan-Meier graph showing that voluntary running does not markedly alter the characteristic short lifespan of mtDNA mutator mice (females, N=7 each group). Significances were determined by two-way ANOVA with posthoc analysis (A, B) or Mantel–Cox test (C), with α $P<0.10$ and ****$P<0.0001$.

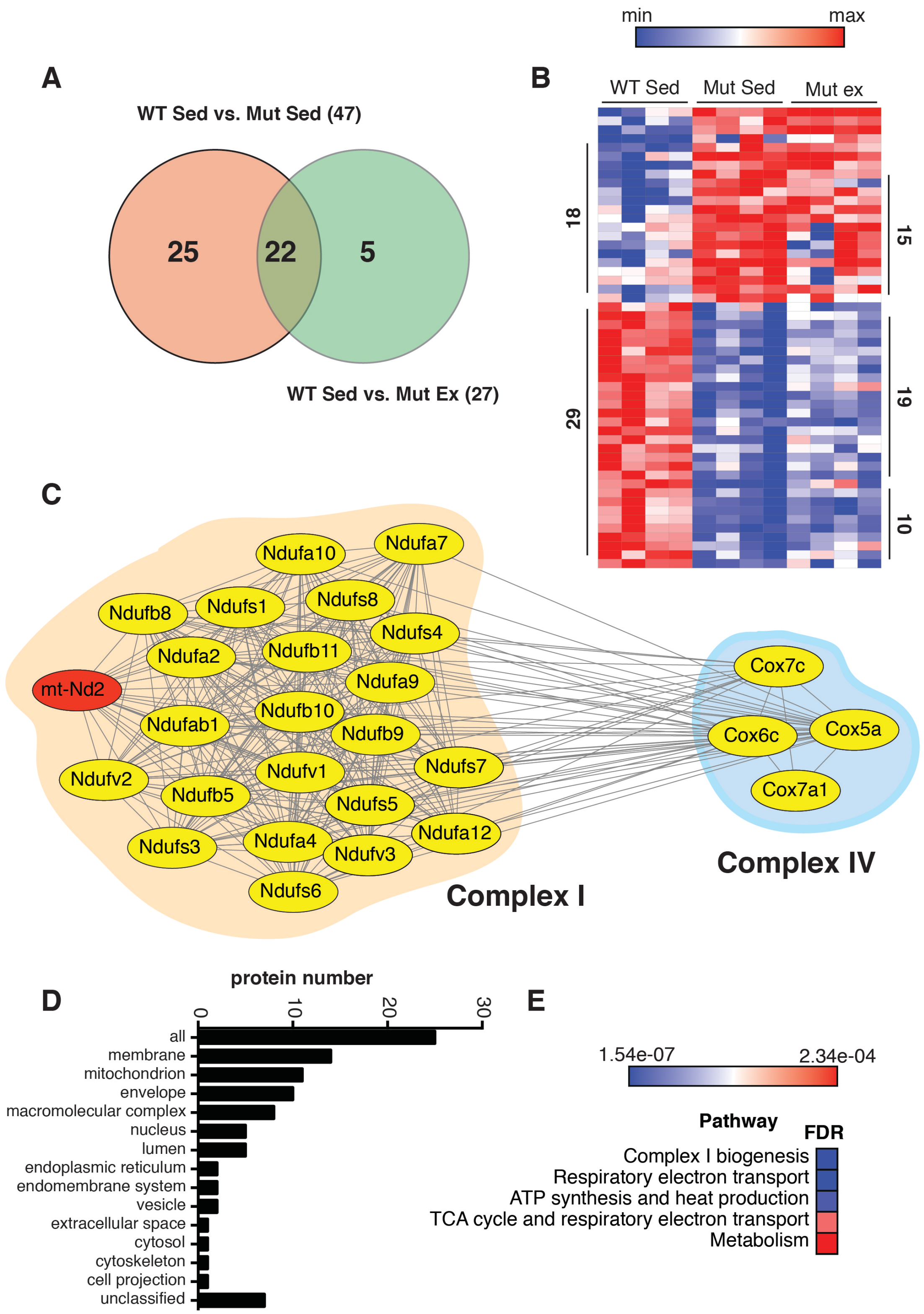

Fig. S4. Proteomic profiles in motor cortex. (A) Venn diagram of proteomic analysis shows significantly fewer altered proteins in motor cortex than in striatum and muscle when comparing 30 week-old sedentary WT and mtDNA mutator mice (47), and exercising mtDNA mutator mice (27) (males, N=4 for each group). Twenty-five proteins were specific to genotype and 5 proteins

were specific to exercising, with 22 proteins dysregulated under both conditions. (B) Heat-map depicting the up- and down-regulation of proteins (18, 29; respectively) in sedentary mtDNA mutator mice, as compared with WT littermates. Exercise rescued 15 of the 18 up-regulated and 10 of the 29 down-regulated proteins, and caused the up-regulation of 4 and the down-regulation of 1 protein. (C) STRING network analysis represented using the Cytoscape platform showing the specific subunits (red for mitochondrial-encoded; yellow for nuclear-encoded) of the OXPHOS complexes I and IV that were down-regulated in mtDNA mutator mice. (D) Gene Ontology Cellular Component analysis of proteins normalized by exercise (25) in the mtDNA mutator mouse. (E) KEGG pathway enrichment analysis of normalized proteins in mtDNA mutator mice reveals complex I biogenesis and respiratory electron transport as top biological processes improved by exercise in the mtDNA mouse cortex. FDR: False discovery rate.

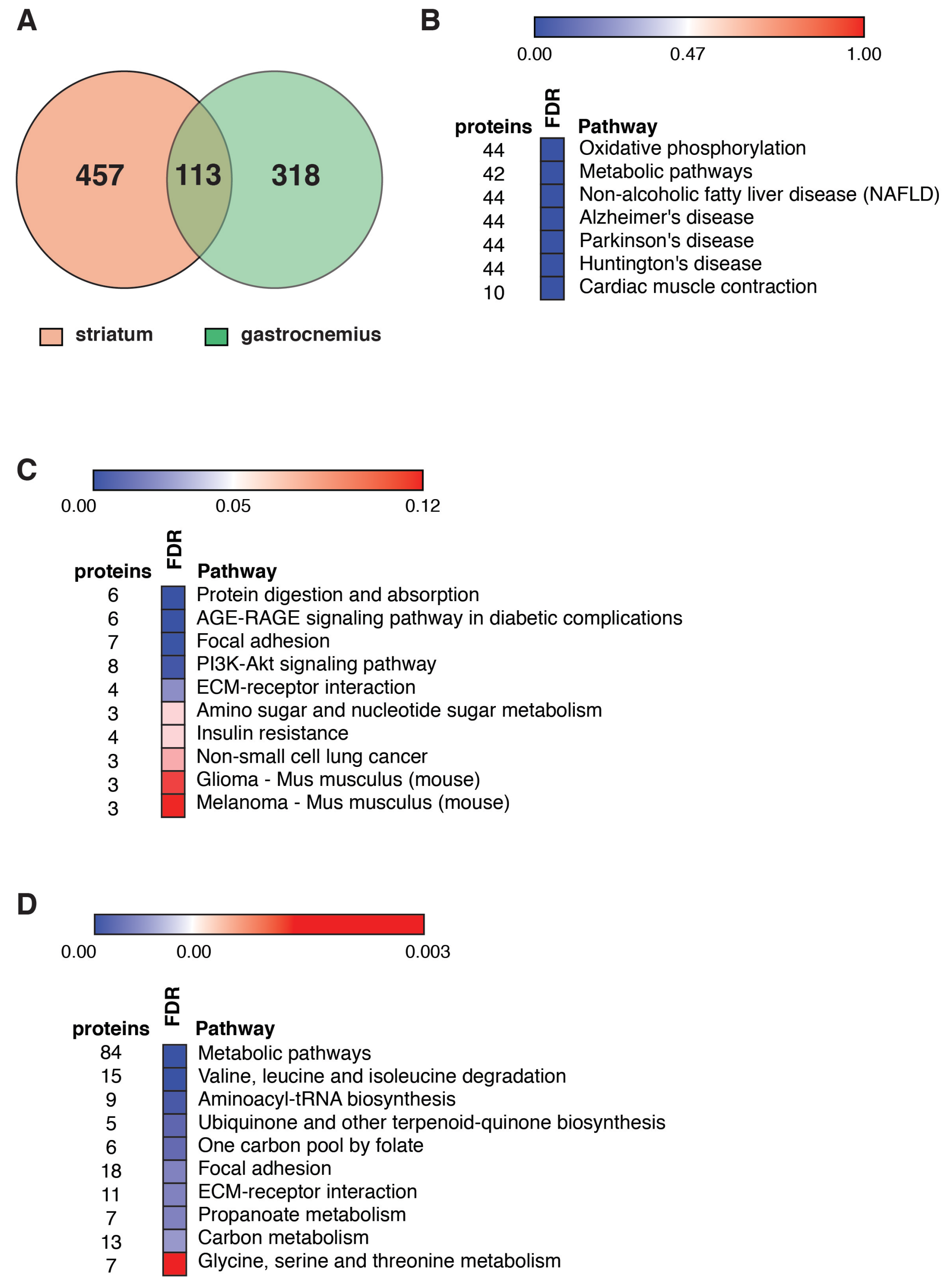

Fig. S5. Comparisons of proteomic profiles in muscle and striatum. (A) Venn diagram representing common proteins deregulated in striatum and gastrocnemius skeletal muscle when comparing 30 week-old sedentary WT and mtDNA mutator mice (males, N=4 for each group). Four hundred and fifty-seven proteins were specific to striatum, 318 to skeletal muscle, and 113 proteins were deregulated in both tissues. (B) KEGG pathway analysis of the 113 common deregulated proteins reveals oxidative phosphorylation as a top affected pathway. (C, D) KEGG pathway analysis of up-regulated proteins in muscle and striatum, respectively. FDR: False discovery rate.

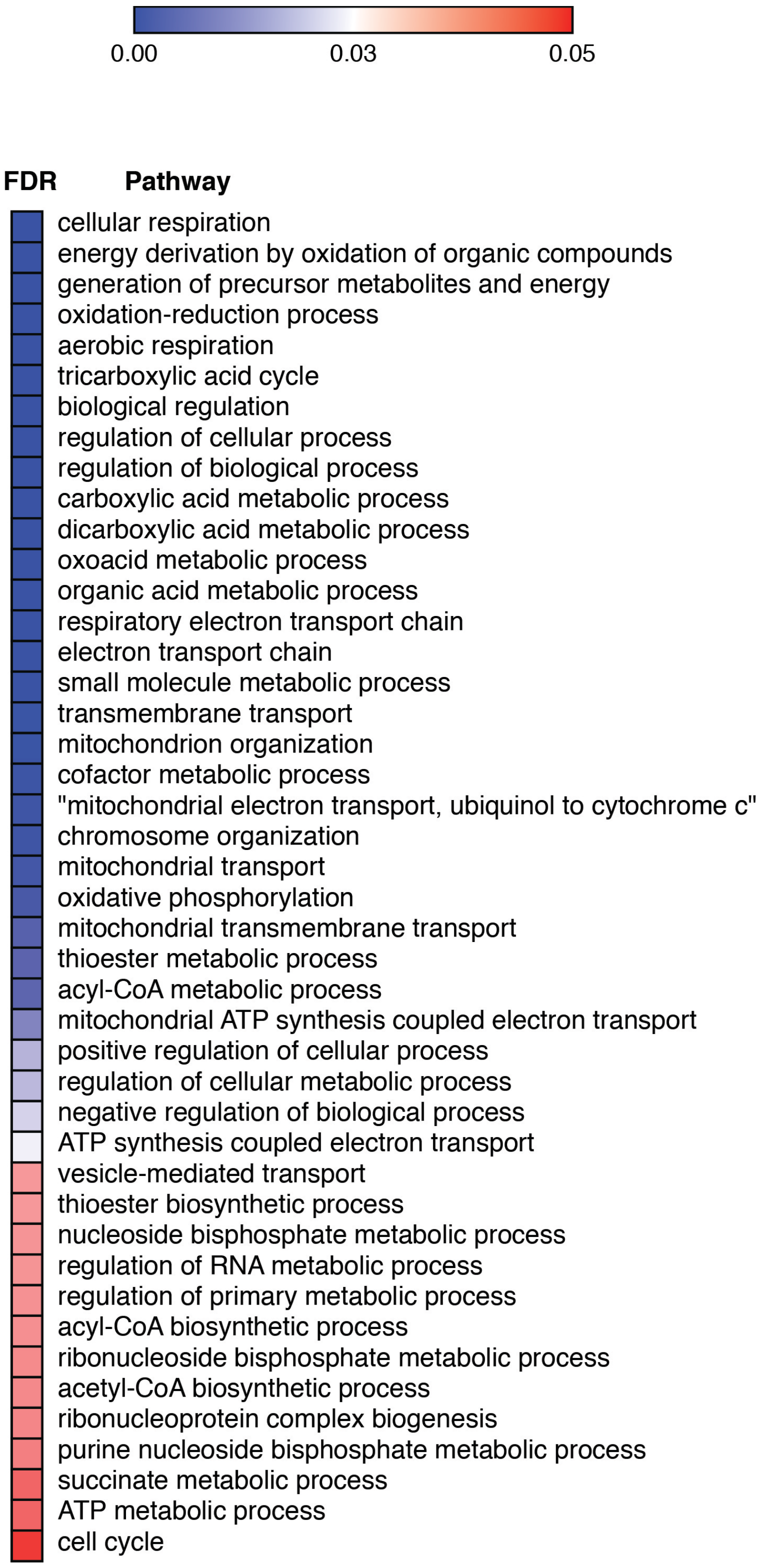

Fig. S6. GO enrichment pathway analysis in muscle. Gene Ontology (GO) term enrichment pathway analysis of normalized proteins in gastrocnemius skeletal muscle when comparing 30 week-old sedentary and exercising mtDNA mutator mice (males, N=4 for each group), with using the total list of identified proteins as a background dataset. FDR: False discovery rate.

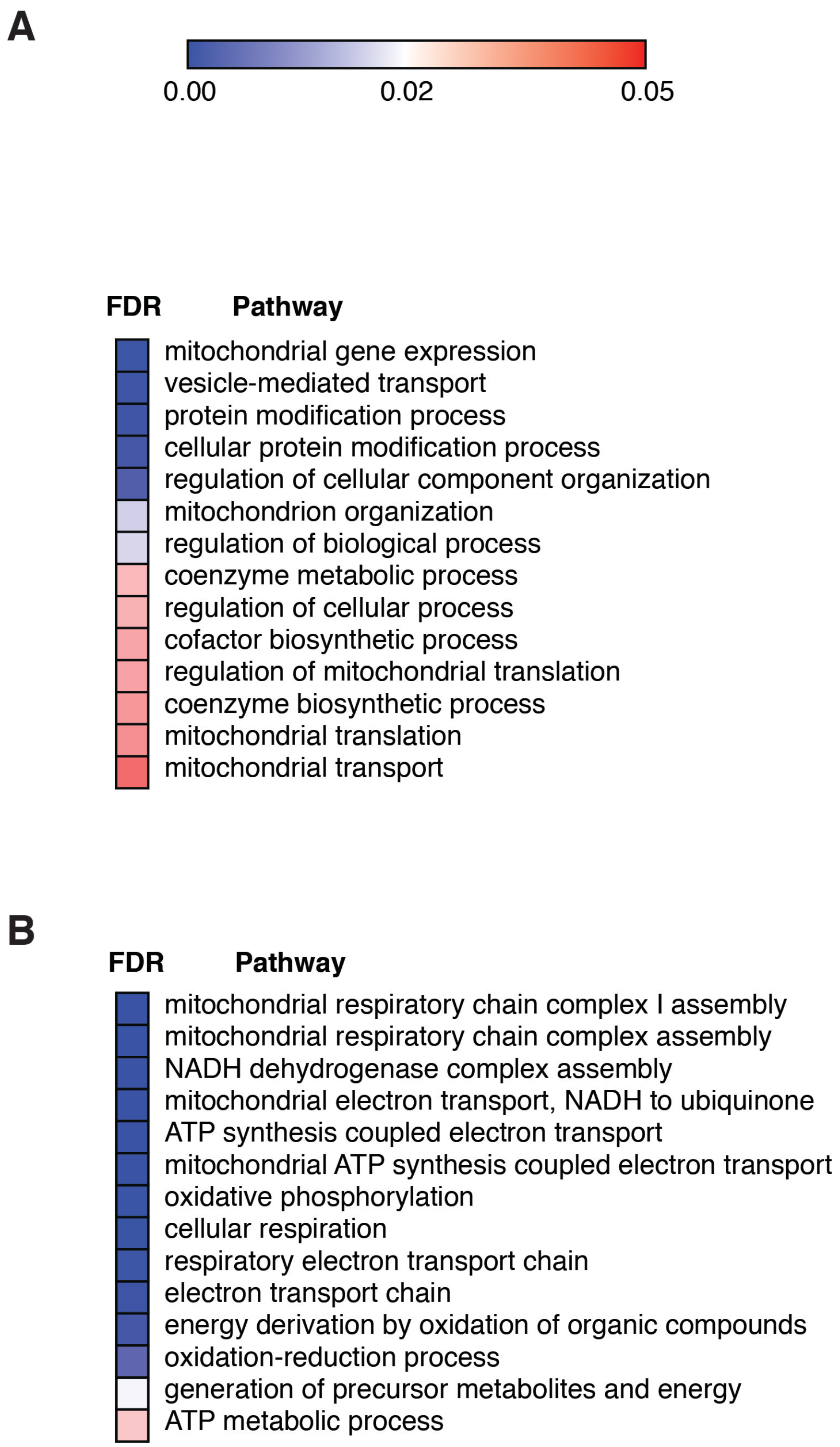

Fig. S7. GO enrichment pathway analysis in striatum and motor cortex. Gene Ontology (GO) term enrichment pathway analysis of normalized proteins in (A) striatum and (B) motor cortex when comparing 30 week-old sedentary and exercising mtDNA mutator mice (males, N=4 for each group), with using the total list of identified proteins as a background dataset. FDR: False discovery rate.

Table S1. TMT sample arrays

Set 1	Sample
**TMT_126**	WT Sed 1
**TMT_127N**	WT Sed 2
**TMT_127C**	WT Ex 1
**TMT_128N**	WT Ex 2
**TMT_128C**	Mut Sed 1
**TMT_129N**	Mut Sed 2
**TMT_129C**	Mut Ex 1
**TMT_130N**	Mut Ex 2
**TMT_130C**	IS
**TMT_131**	IS

Set 2	Sample
**TMT_126**	WT Sed 3
**TMT_127N**	WT Sed 4
**TMT_127C**	WT Ex 3
**TMT_128N**	WT Ex 4
**TMT_128C**	Mut Sed 3
**TMT_129N**	Mut Sed 4
**TMT_129C**	Mut Ex 3
**TMT_130N**	Mut Ex 4
**TMT_130C**	IS
**TMT_131**	IS

IS= Internal Standard was generated by pooling equal amounts of all samples at the peptide level.
